# *Drosophila melanogaster* miPEP8 Regulates Cell Size Through its Interaction With ref(2)P/p62

**DOI:** 10.1016/j.mcpro.2026.101544

**Published:** 2026-02-28

**Authors:** Carine Duboé, Clémence Guillon, Nathanael Jariais, Jessica Boutet, Carole Pichereaux, Karima Chaoui, Emmanuelle Näser, Jean-Philippe Combier, Christine Brun, Yvan Martineau, Odile Burlet-Schiltz, Serge Plaza, Bertrand Fabre

**Affiliations:** 1 Laboratoire de Recherche en Sciences Végétales (LRSV), CNRS/Université de Toulouse/INPT, Auzeville-Tolosane, France; 2 Institut de Pharmacologie et de Biologie Structurale (IPBS), CNRS, UPS, Université de Toulouse, Toulouse, France; 3 Fédération de Recherche (FR3450), Agrobiosciences, Interactions et Biodiversité (AIB), CNRS, Toulouse, France; 4 Infrastructure Nationale de Protéomique, ProFI, FR 2048, Toulouse, France; 5 TAGC (UMR1090), Aix-Marseille Université, INSERM, Turing Centre for Living Systems, Marseille, France; 6 CNRS, Marseille, France; 7 Centre de Recherche en Cancérologie de Toulouse (CRCT), INSERM U1037, Université Toulouse III-Paul Sabatier, ERL5294 CNRS, Toulouse, France; 8 Equipe Labellisée Ligue Contre Le Cancer, Université de Toulouse, Toulouse, France

**Keywords:** microproteins, miPEPs, *Drosophila melanogaster*, cell size, proteomics

## Abstract

MiPEPs are microproteins encoded by primary transcripts of microRNAs (pri-miRNAs). Initially identified in plants, we recently characterized a miPEP in *Drosophila melanogaster*, named miPEP8, which is involved in the regulation of wing size. However, mechanisms at play are unknown. In the present study, we take advantage of the *Drosophila* cell line Schneider 2 (S2) to further investigate miPEP8 function at the molecular level. Overexpressing miPEP8 in S2 cells induced a reduction of cell size as well as an increase of the proportion of cells in the G1 phase of the cell cycle and a decrease of the autophagic flux. A proteomics analysis revealed that miPEP8 overexpression in S2 cells induces the upregulation of several proteins including the autophagosome cargo protein ref(2)P (the orthologue of the human p62/Sequestosome 1 protein). The interactome of miPEP8 was generated and revealed interactions between this miPEP8 and the mTORC1/autophagy pathway. Bioinformatics analysis identified a short linear motif (SLiM) on miPEP8 sequence. Mutation of this SLiM prevented the interaction between ref(2)P/p62 and miPEP8. Mutation of the SLiM also reverted the smaller cell size phenotype observed when overexpressing miPEP8 in S2 cells. RNA interference targeting ref(2)P/p62 reversed the cell size phenotype, suggesting that this protein plays a role in the regulation of cell size in *Drosophila*. Finally, the cell size phenotype was also observed *in vivo* on wings of flies either mutated or overexpressing miPEP8.

## Highlights

• Overexpression of miPEP8 in *Drosophila* S2 cells induces a reduction in cell size.
• miPEP8 interacts with ref(2)P via a short linear motif.
• The regulation of cell size induced by miPEP8 seems to be carried out through its interaction with ref(2)P.
• *In vivo* experiments confirm the role of miPEP8 in cell size regulation.

## Graphical Abstract

**Figure:**
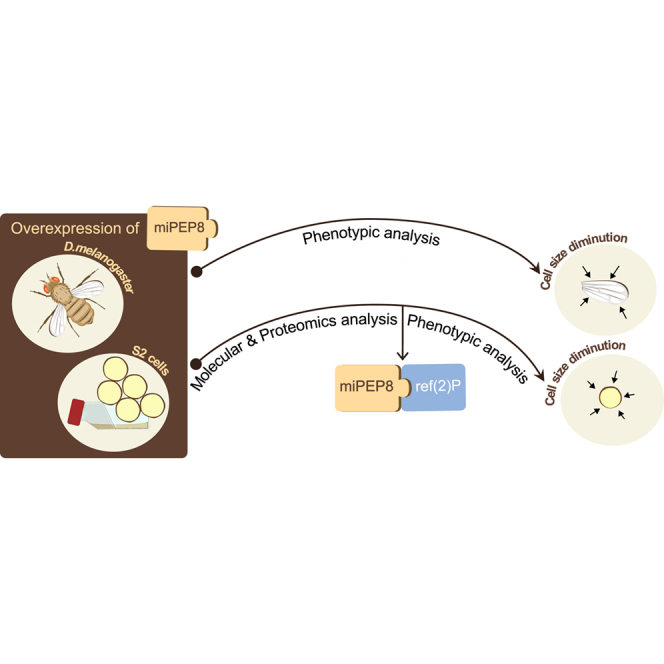

## In Brief

In the present study, we investigate the function of miPEP8 from *Drosophila melanogaster*. Overexpressing miPEP8 in S2 cells induced a reduction of cell size. The interactome of miPEP8 revealed an interaction between miPEP8 and ref(2)P/p62. RNA interference targeting ref(2)P/p62 reversed the cell size phenotype, suggesting that this protein plays a role in regulating cell size in *Drosophila*. Finally, the cell size phenotype was also observed *in vivo* on wings of flies either mutated or overexpressing miPEP8.

The discovery of the pervasive translation of short open reading frames (sORFs) into thousands of microproteins has revolutionized our view of the proteome (1, 2). These small (typically less than 100 amino acids) unannotated proteins are produced from a variety of RNAs in all organisms from the kingdom of life (3, 4, 5, 6, 7, 8, 9, 10). Microproteins have appeared as important regulators of key cellular processes (11). Although thousands of microproteins have been identified across many model organisms, we still lack a comprehensive characterization for most of their functions (11). One of the organisms that pioneered the study of microproteins is *Drosophila melanogaster*, notably through the discovery of the function of the Pgc and Pri/Tal peptides (12, 13, 14, 15, 16). Although the proteome of *D. melanogaster* is well characterized (17, 18, 19, 20, 21, 22, 23), the discovery of its microproteome is still in its infancy (3, 15, 24, 25, 26). Recent studies in this model organism show that many microproteins can be produced from the translation of sORFs in alternative reading frames within canonical ORFs (3, 24), or that some of them are encoded by microRNA (*miR*) genes (3, 27, 28). These microproteins produced from *miR* genes are called miPEPs (miRNA-encoded peptides) and were first discovered in plants (29). Since their discovery, it was shown that miPEPs are present in a variety of plants but also in animals (30), although they seem to carry different functions in these two kingdoms (30, 31). In plants, miPEPs were shown to increase the expression level of their corresponding pri-miRNAs (29). In animals, however, miPEPs play different molecular roles, likely through interaction with different proteins, albeit in some cases affecting similar pathways than their corresponding miRNAs (30, 32).

Our group recently identified a microprotein encoded by *miR-8* of *D. melanogaster*, named miPEP8 (28). *MiR-8* was shown to regulate body size in *Drosophila*, including wings (33). This is mediated through the binding of the *miR-8* miRNA to the mRNA coding for the U-shaped (USH) protein, an inhibitor of the phosphoinositide-3 kinase (PI3K) activity (33). Our previously published data shows that miPEP8 is also involved in the regulation of wing size in flies, albeit with reduced phenotype compared to *miR-8* mutants (in which both the miRNA stem loop and the miPEP8 ORF are deleted) (28, 33). Interestingly, both the overexpression and mutation of miPEP8 induce a reduction in wing size in flies (28), suggesting that the correct dosage is necessary within cells. Overexpression of miPEP8 results in a lower allelic frequency ratio compared to controls, indicating potential issues with fecundity and viability, and suggesting the involvement of miPEP8 in fitness, a marker/phenotype used in population genetics for a long time that has emerged as a sensitive detector of gene function (34, 35). The molecular mechanisms involved in the reduction of wing size upon miPEP8 overexpression or deletion are still not elucidated (28). In the present study, we use the *Drosophila* cell culture line Schneider 2 (S2) to further investigate miPEP8 function at the molecular level. Overexpressing miPEP8 in S2 cells induced a reduction of cell size, in agreement with the phenotypes observed in flies, as well as an increase in the proportion of cells in the G1 phase of the cell cycle and an increase in the autophagic flux. A mass spectrometry-based proteomics approach revealed that overexpressing miPEP8 in S2 cells induces the upregulation of several proteins including the autophagosome cargo protein ref(2)P (the orthologue of the human p62/Sequestosome 1 protein). In order to gain some insight into the mode of action of miPEP8, its interactome was generated and revealed interactions between this microprotein and the mTORC1 (mechanistic target of rapamycin complex 1)/autophagy pathway. Bioinformatics analysis identified a short linear motif (SLiM) on miPEP8 sequence. Mutation of this SLiM prevented the interaction between miPEP8 and several proteins, including ref(2)P/p62. Mutation of the SLiM also reverted the smaller cell size observed upon overexpression of miPEP8 in S2 cells. Finally, the small cell size phenotype was also abolished when cells were treated with RNA interference targeting ref(2)P, suggesting that this protein is involved in the function of miPEP8 in regulating S2 cell size in *Drosophila*. The effect of the overexpression (or deletion) of miPEP8 on cell size was recapitulated *in vivo* on flies’ wings. Altogether, our data points toward a function of miPEP8 in the regulation of cell size of *Drosophila* through its interaction with the autophagosome cargo protein ref(2)P.

## Experimental Procedures

### S2 Cell Culture

S2 cells were cultured as described previously (28).

### Confocal Microscopy

For imaging experiments, S2 cells were co-transfected using an actin-GAL4 driver with UAS-EGFP, UAS-EGFP-miPEP8, UAS-EGFP-miPEP8 SLiMmt (mutation of the MOD_Plk1/MOD_Plk4 SLiM identified on miPEP8, the “REKSIL” amino acids were modified to “AAAAAA”) or UAS-miPEP8. S2 cells were transfected with Effectene (Qiagen) as described in (28). Forty-eight hours after transfection, the cells were prepared for microscopy analysis as previously described (3). Briefly, cells were transferred onto coverslips, fixed using 4% paraformaldehyde (Sigma-Aldrich) in phosphate-buffered saline (PBS) for 20 min at 20°C, washed twice with PBS, and mounted with ProLong Diamond Antifade Mountant (Invitrogen). Slides were analyzed on a SP8 Leica confocal microscope.

For LysoTracker experiments, viable cells were incubated on coverslips for 20 min in complete Schneider’s Insect Medium with 50 nM LysoTracker (ThermoFisher Scientific). Cells were washed with PBS before fixation and further fixed and mounted as described above.

For three-dimensional (3D) analysis, cell volume was measured in μm^3^ using the ImageJ software (36). Confocal z-stacks were processed using the 3D Segmentation plugin. Regions of interest (ROIs) corresponding to individual cells were segmented based on the EGFP signal, or on the DAPI signal for the comparison of transfected and non-transfected cells. The plugin was used to reconstruct cell volumes from z-stacks. Measurements were exported from the 3D ROI Manager and normalized to the mean volume of EGFP-expressing cells for quantitative comparison between samples. In LysoTracker-treated cells, the signal intensity was measured in the 3D construction using Integrated Density ("RawIntDen", sum of the values of the pixels in the selection) and divided by the corresponding cell volume.

### Cell Cycle Analysis

S2 cells transfected with UAS-EGFP or UAS-EGFP-miPEP8 were treated for 24h either with 1 μM Hydroxyurea +10 μM Aphidicolin, 1.7 μM 20 Hydroxyecdysone or 2.7 μM Colcemid to synchronize cells respectively in S, G2 and M phase. The G1 phase was obtained by subtracting the areas of the other phases of the cell cycle.

For flow cytometry experiments, S2 cells were collected in 15 ml tube. They were fixed in 1% formaldehyde on ice for 30 min. Then the cells were permeabilized in PBT (phosphate buffer saline + 0.1% Tween-20) at room temperature for 5 min. Nucleus were stained by adding 1 μg/ml DAPI to the cell suspension for 30 min in the dark. Cell were then sorted using a LSRFortessa X-20 flow cytometer (BD Biosciences) and analysis was performed using the Flowjo V10 software (BD Biosciences).

### Proteome Analysis

S2 cells were co-transfected using an actin-GAL4 driver with UAS-EGFP or UAS-EGFP-miPEP8 before cell sorting. Cells were fixed in 1% formaldehyde on ice for 30 min, permeabilized in PBT at room temperature for 5 min and nucleus stained with 1 μg/ml DAPI for 30 min. Then cells were sorted using a FACSAria Fusion flow cytometer (BD Biosciences). After cell sorting, cells were lysed in an SDS-based buffer (Tris 50 mM pH 7.5, 5% SDS) and BCA assay (Thermo Scientific) was performed. 10 μg of proteins were loaded on SDS-PAGE and in-gel digestion was performed as previously described (37). The resulting peptides (50 ng) were analyzed by nanoLC-MS/MS using an UltiMate 3000 RS nanoLC system (ThermoFisher Scientific) coupled to a TIMS-TOF SCP mass spectrometer (Bruker). Peptides were separated on a C18 Aurora column (25 cm × 75 μm ID, IonOpticks) using a gradient ramping from 2% to 20% of B in 30 min, then to 37% of B in 3 min and to 85% of B in 2 min (solvent A: 0.1% formic acid in H2O; solvent B: 0.1% FA in acetonitrile), with a flow rate of 150 nl/min. MS acquisition was performed in DIA-PASEF mode on the precursor mass range [400–1000] m/z and ion mobility 1/K0 [0.64–1.37]. The acquisition scheme was composed of 8 consecutive TIMS ramps using an accumulation time of 100 ms, with 3 MS/MS acquisition windows of 25 Th for each of them. The resulting cycle time was 0.96 s. The collision energy was ramped linearly as a function of the ion mobility from 59 eV at 1/K0 = 1.6 Vs cm−2 to 20 eV at 1/K0 = 0.6 Vs cm−2. Resulting.d files were analyzed using DIANN version 2.0 (38). The maximum number of missed cleavages was set to 1, the mass tolerance for precursor and fragment ions was set to 10 ppm and 14 ppm, respectively. The number of maximum variable modifications (oxidation of methionine and acetylation of protein N-terminus) was set to 1. Carbamidomethylation of cysteines was set as a fixed modification. Precursor charge range were set from 2 to 4, precursor m/z range was set from 400 to 1000, fragment ion m/z range was set from 100 to 1700 and cross-run normalization was set to global. A database containing proteins from Uniprot (July 2020, 42,675 sequences), miPEP8 and EGFP sequences and common contaminants was used. A false discovery rate was set to 1% for both peptide and protein levels. Proteins with a Log_2_ fold change < −1 or >1 and a *p*-value from a welch *t* test <0.05 were considered differentially regulated.

The Western blot analysis validating the increased level of ref(2)P upon miPEP8 overexpression in S2 cells was performed using anti-ref(2)P (Abcam), anti-GAPDH (39) and anti-GFP antibodies (Cell Signaling Technology).

### Interactome Analysis

S2 cells were co-transfected using an actin-GAL4 driver with UAS-EGFP, UAS-EGFP-miPEP8 or UAS-EGFP-miPEP8 SLiMmt and harvested 48h after transfection. Cells were lysed in a buffer containing 50 mM Tris pH 7.5, 50 mM NaCl, 5% glycerol, 0.1% Igepal and protease inhibitor cocktail (Sigma Aldrich) and immunoprecipitation was performed using 40 μl of slurry of ChromoTek GFP-Trap Agarose beads (ChromoTek GmbH, Planegg-Martinsried, Germany) O/N at 4 °C. Beads were washed four times in lysis buffer and boiled in 50 μl of 2X Laemmli for 10 min at 100 °C. Proteins were then alkylated using 60 mM of chloroacetamide and in gel digestion was performed as previously described (37). The resulting peptides were injected either on a nanoRS UHPLC system coupled to a Thermofisher LTQ Orbitrap Velos or a Thermofisher Q Exactive Plus as previously described (40). Raw data were analyzed using MaxQuant version 1.6.14.0 (41) using default parameters and the same database as for proteome analysis. Briefly, the maximum number of missed cleavages was set to 2, the mass tolerance for the precursor was set to 20 and 4.5 ppm for the first and the main searches, respectively, and 20 ppm for the fragment ions. Oxidation of methionine and acetylation of protein N-terminus were set as variable modifications. Carbamidomethylation of cysteines was set as a fixed modification. A false discovery rate was set to 1% for both peptide and protein levels. To determine the miPEP8 interactome, a ratio was calculated for each protein between the minimal intensity value measured among the EGFP-miPEP8 immunoprecipitation (IP) replicates and the maximal intensity value measured among the EGFP IP replicates as described in (42). The minimum ratio threshold to define a protein as a miPEP8 interacting partner was defined as previously described (43). Briefly, this threshold corresponds to Q3 + 1.5 × IQ, Q3 being the third quartile and IQ being the interquartile, both based on the distribution of all the Log_2_ ratio (EGFP-miPEP8 IP/EGFP IP) measured for each protein quantified in the experiment. In addition, a minimum ratio (EGFP-miPEP8 IP/EGFP IP) of 2 based on spectral counts was required to consider a protein as a miPEP8 partner. Finally, in the case of proteins present only in miPEP8 IPs (immunoprecipitations) samples, an intensity superior to the Q1 (first quartile, based on the distribution of all the measured intensity) had to be observed in at least one of the miPEP8 IP replicates to consider the protein as a miPEP8 partner.

In the case of the comparison of the interactome of EGFP-miPEP8 SLiMmt and EGFP-miPEP8, proteins were considered differentially associated if they were identified as interactor of miPEP8 (see above) and the Log_2_ ratio of the average intensities of EGFP-miPEP8 SLiMmt on the average intensities of EGFP-miPEP8 was < −1 or >1 and the *p*-value from a welch *t* test <0.05. Gene Ontology terms enrichment and protein networks were generated using String v12.0 (44).

### Experimental Design and Statistical Rationale

All the MS experiments were performed on at least three biological replicates (*n* described in each figure legend) but no technical replicates were performed. To assess statistical differences in protein intensities, welch *t* test were performed in addition to a minimum of 2-fold difference between the compared conditions. For the definition of miPEP8 interactome, we used a stringent analysis in which we generated a ratio was for each protein calculated between the minimal intensity value measured among the EGFP-miPEP8 immunoprecipitation (IP) replicates and the maximal intensity value measured among the EGFP IP replicates as described in (42). This displays the worst possible ratio between the miPEP8 and control IP. Then, we only considered the protein with extreme ratios as interactors candidates (ratio threshold of 9.66). In addition, a minimum ratio (EGFP-miPEP8 IP/EGFP IP) of 2 based on spectral counts was required to consider a protein as a miPEP8 partner.

### miPEP8 interactors validation by co-immunoprecipitation

Plasmids coding for HA-tagged versions of interactors identified by MS as described above (and the GAPDH as a negative control) were obtained from the Berkeley *Drosophila* Genome Project (BDGP) (45). S2 cells were co-transfected co-transfected using an actin-GAL4 driver with with UAS-EGFP, UAS-EGFP-miPEP8 or UAS-EGFP-miPEP8 SLiMmt and the plasmid for each protein interactor (and the GAPDH as a negative control). Immunoprecipitations were performed using of ChromoTek GFP-Trap Agarose beads (ChromoTek GmbH, Planegg-Martinsried) as described above and a western-blot analysis was performed using anti-HA (Sigma-Aldrich) and anti-GFP antibodies (Cell Signaling Technology).

### Split-Luciferase Analysis

For split-luciferase assays, 7 × 10^5^ *Drosophila* S2 cells were seeded in 24-well plates in 500 μl Schneider’s *Drosophila* Medium and cultured for 24 h prior to transfection. Cells were co-transfected with a total of 200 ng of plasmid DNA encoding an actin promoter–driven miPEP8 or Eyeless (Ey) fused on their C-terminus to the N-terminal fragment of luciferase (N-Luc) and the C-terminal fragment of luciferase (C-Luc) fused the N-terminus of the indicated candidate proteins and Antenapedia (Antp). EGFP-expressing plasmid (20 ng) were included in each condition as a transfection control. Transfections were performed using Effectene (Qiagen) according to the manufacturer’s instructions. Experiments were conducted using at least three independent biological replicates. At 48 h post-transfection, cells were collected and resuspended in 500 μl PBS. For each condition, 75 μl of cell suspension was distributed into three technical replicates in a white 96-well plate. Luminescence was measured following the addition of 5 μl of 10 mM D-luciferin, after a 30 min incubation in the dark. Luminescence emission kinetics were recorded using a Victor Nivo plate reader (PerkinElmer) equipped with a 700 nm infrared blocker filter, with an integration time of 1s per well. The luminescence intensity for each condition of each replicate was normalized using the median luminescence intensity of all the conditions within each replicate. EGFP fluorescence was checked using excitation at 480/30 nm and emission at 530/30 nm, with an integration time of 0.5 s per well.

### Plasmids, dsRNAs, and qPCR

The dsRNAs were generated with, respectively, pUFO Rag C/D-HA (DGRC number UFO02654), pUFO Ref(2)P-FLAG-HA (generated by subcloning Ref(2)P sequence, DGRC number FMO06145), and Luciferase ICE T7 control vector (Promega) according to (46) using the following primers as described in the Supplemental Table S1. S2 cells were harvested 48h post-transfection. RNAs were extracted using the RNeasy kit (Qiagen) and reverse transcription were performed using the M-MLV Reverse Transcriptase (Promega) following manufacturer’s instructions.

qPCR analysis was performed using the LightCycler 480 SYBR Green I Master (Roche Life Science) on a LightCycler 480 Instument II (Roche Life Science) and analyzed with the LightCyler 480 Software (Roche Life Science), using the 2-ΔΔCt method (47). A list of the primers used can be found in Supplemental Table S1.

### Short Linear Motif Identification and miPEP8 Interactor Predictions

Short linear motifs (SLiMs) have then been detected in the miPEP8 sequence using the ELM prediction tool of the ELM database (48), considering only the true positive instances detected in *D. melanogaster*.

mimicINT, a workflow initially developed for microbe-host protein interactions inference (49) has been used to infer protein-protein interactions between miPEP8 and the *Drosophila* canonical proteins. Adapted to the sPEP-canonical protein in *Drosophila*, it then consists in (i) the detection of short linear motifs, i.e. SLiMs, extracted from the ELM database (48) and globular domains predicted using the existing InterPro signatures (50) in the sPEP sequence; (ii) the collection of domains on the *Drosophila* canonical proteins; (iii) the interaction inferences between sPEP and canonical proteins, according to interaction templates (see (49) for further details). Notably, only true positive instances of SLiMs detected in *D. melanogaster* are considered for interaction predictions (http://elm.eu.org/instances/?q=&instance_logic=&taxon=Drosophila+melanogaster).

### Detection of Protein Domains on miPEP8 Sequence

InterProScan (v5.52–87.0) (51) was used to look for protein domains on miPEP8 sequence.

### Fly Strains and Genetics

*Drosophila* flies were maintained on standard cornmeal-yeast medium (Dutscher). miPEP8 knock out flies and miPEP8 overexpressing flies experiments were generated and used as described previously (28). Experiments shown are the sum of at least 3 independent crosses. n indicates the number of individuals analyzed. For wing measurements, young flies (2–5 days) of the appropriate genotypes were collected and stored in Ethanol. For analysis of wings, female adult wings were removed in wash buffer (PBS and 0.1% Triton X-100) and mounted on a slide in 80% glycerol in PBS. Wings images were acquired on a Nikon eclipse Ts2 microscope using x4 and x20 magnification. Measurements of wing size were performed on captured digitalized images using IMAGE J software.

Since epidermal cells on the *Drosophila* wing produce a single cuticular hair (52), wing cell numbers and cell size were calculated by counting the number of hairs per surface area between the L3 and L4 veins according to (33, 53). Changes in the size of intervein cells were calculated by counting the number of hairs (the base of each hair must be visible for the hair to be included) located between veins 3 and 4 of the wing blade in a square of 0.01 mm^2^.

## Results and Discussion

### Overexpression of miPEP8 in S2 cells Decreases Cell Size, Accumulates Cell in G1 Phase, and Changes the expression of a Set of Proteins

**Fig. 1:**
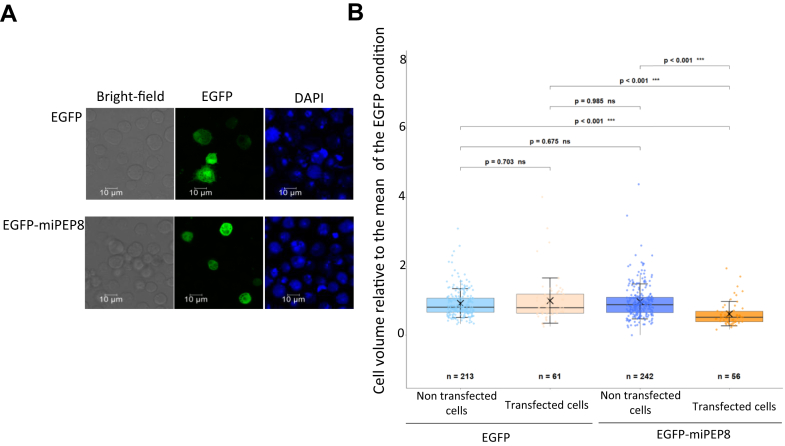
**Overexpression of miPEP8 decreases S2 cell size.***A*, 2 cells were transfected with a plasmid encoding either the EGFP or EGFP fused to miPEP8 (EGFP-miPEP8). Cells were stained with DAPI and observed by confocal microscopy. *B*, the size of the S2 cells expressing either EGFP or EGFP-miPEP8 was measured and normalized to the mean of the measured EGFP cell size. The *p*-values were calculated using a one-way ANOVA (Analysis of Variance), and for groups comparison, a Tukey's HSD (Honestly Significant Difference) test was performed (n = 213, 61, 242 and 56 for EGFP non-transfected, EGFP-transfected, EGFP-miPEP8 non-transfected, and EGFP-miPEP8-transfected cells, respectively, from 3 independent experiments).

To gain some insights into the molecular function of miPEP8, we used Schneider 2 (S2) cells as it is one of the most used cell lines for studies in *Drosophila*. MiPEP8 was not detected in this cell line (28); thus, we decided to work with the overexpression of this microprotein in S2 cells. Upon overexpression of an EGFP-miPEP8 (Enhanced Green Fluorescent Protein-miPEP8) fused protein, we observed a decrease in S2 cell size (*p* < 0.001) (Fig. 1, *A* and *B*). The median size of cells overexpressing EGFP-miPEP8 was 26.8% smaller than the median size of cells overexpressing EGFP (Fig. 1*B*). This diminution of cell size is in agreement with previous data showing that overexpressing miPEP8 in *D. melanogaster* wings induces a decrease in their size (28). Importantly, this decrease in cell size was not observed in the cells that were not transfected (*i.e.* cells not expressing any EGFP from the same experiment) (Fig. 1*B*), showing that the observed phenotype is specific to cells expressing EGFP-miPEP8. To avoid any artifacts linked to the fusion of miPEP8 (71 amino acid long) to an EGFP protein, we monitored the effect of the transfection of a plasmid expressing a version of miPEP8 not fused to any tag on cell size. Overexpression of miPEP8 promoted a decrease in S2 cell size (*p* < 0.001) similar to the one induced by the overexpression of EGFP-miPEP8 (*p* < 0.001) (Supplemental Fig. S1), showing that miPEP8 retains its activity even fused with a bigger protein (EGFP). Flow cytometry analysis showed that overexpression of miPEP8-EGFP accumulates cells in the G1 phase of the cell cycle compared to cells overexpressing EGFP (*p* = 2.9E-02) or non-transfected cells (*p* = 3.4E-03) (Supplemental Fig. S2).

**Fig. 2:**
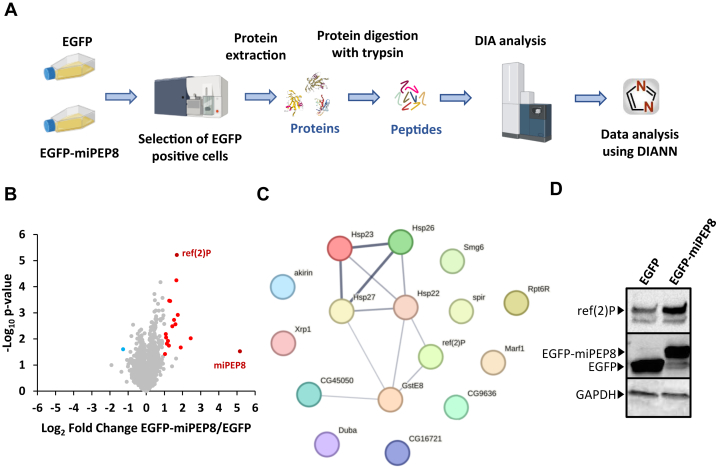
**Data independent analysis proteomics workflow to identify proteins differentially regulated in S2 cells upon overexpression of miPEP8.***A*, S2 cells were transfected with EGFP or EGFP-miPEP8 (n = 3 each) and the cells expressing the fluorescent protein were selected by fluorescence-activated cell sorting. Proteins were extracted and digested with trypsin and resulting peptides were run in data-independent acquisition mode on a TIMS-TOF SCP mass spectrometer (Bruker). The resulting.d files were analyzed with DIANN. *B*, Volcano plot representing the log_2_ ratio (EGFP-miPEP8/EGFP) for each protein quantified and the corresponding *p*-value obtained from a Welch's *t* test. The *blue*, *red*, and *gray dots* represent the proteins more abundant in the cells expressing EGFP, more abundant in the cells expressing EGFP-miPEP8, or not differentially expressed between the two conditions, respectively. The protein ref(2)P/p62 is displayed in *black* on the volcano plot. *C*, STRING network of the proteins more abundant in the EGFP-miPEP8 condition. The line thickness between two proteins indicates the strength of data support. *D*, Western blot analysis for ref(2)P/p62, GFP and GAPDH were performed on the lysates of S2 cells expressing EGFP or EGFP-miPEP8.

The expression of miPEP8 in S2 cells induces a phenotype, albeit discrete, on cell size and cell cycle (Fig. 1, *A* and *B*, Supplemental Figs. S1 and S2). Thus, we decided to monitor how the overexpression of miPEP8 alters the proteome of S2 cells. S2 cells were transfected with either EGFP as a control or EGFP-miPEP8. Cells expressing either protein were sorted using fluorescence-activated cell sorting (FACS), lysed, and proteins were digested with trypsin and analyzed in data-independent acquisition (DIA) on a TIMS-TOF SCP mass spectrometer (Bruker) (Fig. 2*A*). Five thousand one hundred thirty-six proteins were quantified, 18 proteins being differentially regulated between EGFP and EGFP-miPEP8 expressing cells (1 more abundant in EGFP and 17 more abundant in EGFP-miPEP8 expressing cells) (Fig. 2*B* and Supplemental Table S2). Among the proteins up-regulated (Fig. 2*C*) we could find an enrichment of proteins involved in cellular response to stress (*p* = 2.5E-02) and more particularly cellular response to heat stress (p = 6E-04) (Supplemental Fig. S3). This set of proteins includes small heat shock proteins (Hsp22, Hsp23, Hsp26 and Hsp27), the expression of which is developmentally regulated and involved in a wide range of function (21, 54). In addition, the Atg18b (autophagy-related gene 18b) and ref(2)P (refractory to sigma P) proteins, both involved in autophagy (55), were also found to be increased following overexpression of EGFP-miPEP8 (Fig. 2, *B* and *C*). This increase in protein expression was confirmed for ref(2)P by Western blot (Fig. 2*D*). Reanalyzing previously published RNA-seq data (28), we could observe an increase of the mRNA levels of the small heat shock proteins (Hsp22, Hsp23, Hsp26, and Hsp27) upon overexpression of miPEP8 but not for ref(2)P, suggesting a post-transcriptional mechanism for the regulation of this protein (Supplemental Fig. S4). In summary, this proteomics analysis points towards a possible role of miPEP8 in the regulation of cellular stress.

### Defining miPEP8 Interactome in S2 Cells Links This Microprotein With the Intracellular Signaling and Autophagy Pathways

**Fig. 3:**
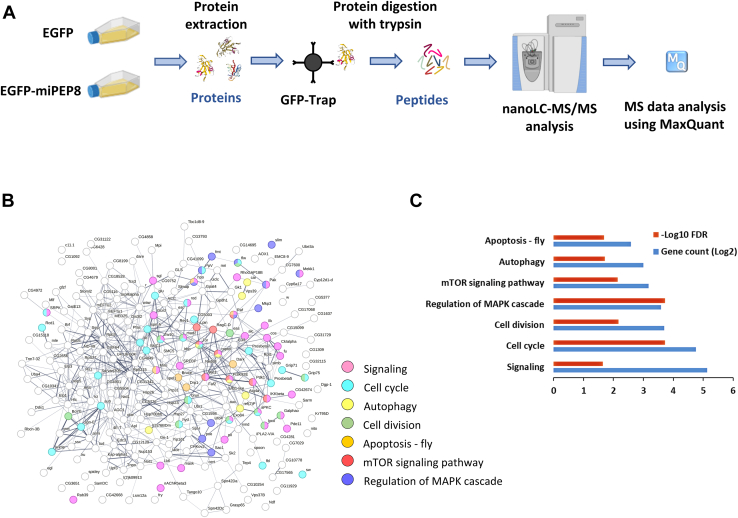
**The interactome of miPEP8 reveals its interaction with a wide range of cellular pathways.***A*, S2 cells were transfected with EGFP or EGFP-miPEP8 (n = 3 for EGFP-miPEP8 and n = 4 for EGFP) and proteins were extracted and subjected to a GFP-Trap agarose beads (Chromotek) precipitation. After elution, proteins were concentrated in one band on SDS-PAGE and digested with trypsin and resulting peptides were run in data-dependent acquisition mode on a Q Exactive plus mass spectrometer (Thermofisher). The resulting raw files were analyzed with MaxQuant. *B*, STRING network of the proteins found as interacting with EGFP-miPEP8. The proteins belonging to the pathways found enriched by STRING were displayed with different colors. The line thickness between two proteins indicates the strength of data support. *C*, GO term analysis using STRING for the proteins interacting with miPEP8. The number of proteins identified in each pathway (gene count, log2 transformed) and the corresponding *p*-value (−log10 transformed) are represented on the graphs.

Given that the overexpression of miPEP8 impacts cell size and induces the expression of proteins involved in cellular stress response, the interactome of this microprotein was generated to identify its potential protein partners (Fig. 3*A*). Either the EGFP or the EGFP-miPEP8 fusion was overexpressed in S2 cells and, after cell lysis, an affinity purification was performed using GFP-Trap agarose beads (Fig. 3*A*). Following protein digestion, peptides were analyzed by LC-MS/MS (Fig. 3*A*) and stringent criteria were used to consider a protein as a miPEP8-interacting protein (see methods section). Despite these stringent criteria, 211 proteins were found interacting with miPEP8 (Fig. 3*B* and Supplemental Table S3), suggesting that miPEP8 might be involved in several cellular pathways (Fig. 3, *B* and *C*). Among the most represented pathways, Signaling, Cell cycle, Cell division, Regulation of MAPK cascade, mTOR signaling, Autophagy and Apoptosis were identified (Fig. 3, *B* and *C*). All these pathways were shown to affect cell size in some capacity (56, 57, 58). Several of these pathways, namely mTOR, apoptosis, and autophagy, are linked to the cellular stress response, as observed in the proteome of miPEP8 overexpressing cells (Supplemental Fig. S3).

**Fig. 4:**
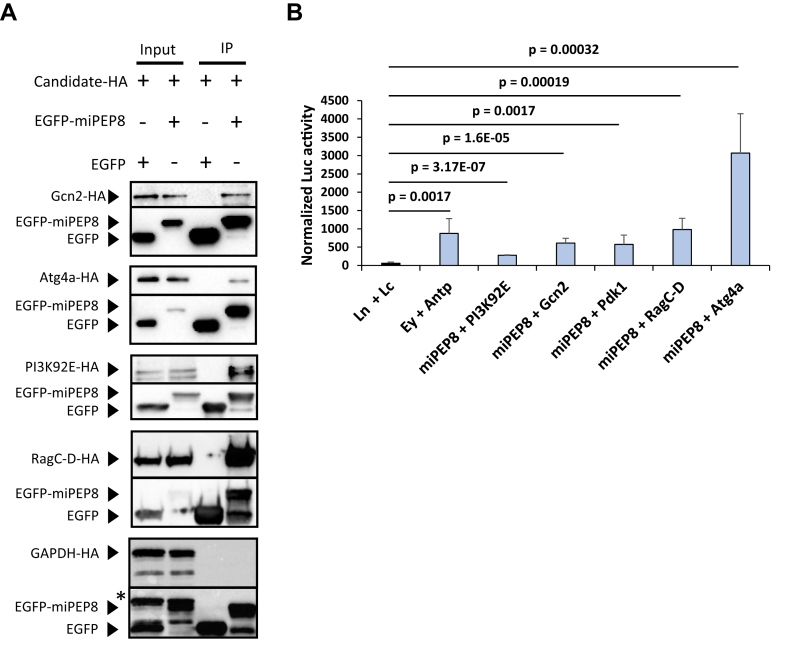
**Validation of the interaction between miPEP8 and proteins involved in the autophagy and intracellular signaling pathways.***A*, co-immunoprecipitation experiments were performed after lysis of S2 cells transfected with plasmids encoding miPEP8 interacting candidates fused with a HA tag and plasmids encoding either EGFP or EGFP-miPEP8 (n = 3 for each candidate). Western blot analysis for HA and GFP were performed. *B*, S2 cells were transfected with plasmids expressing miPEP8 fused with the N-terminal fragment of the luciferase and each protein candidate fused with the C-terminal fragment of the luciferase. In the case of addition of a competitor (Comp), S2 cells were also transfected with a plasmid encoding the corresponding candidate fused with a HA tag (and not fused to the C-terminal fragment of the luciferase). The N-terminal (Ln) and C-terminal (Lc) of the luciferase not fused to any proteins were used as negative control. The proteins eyeless (Ey) and Antenapedia (Antp) were used as positive control of interacting proteins. The *p*-value was calculated using a Welch's *t* test (n = 7 for each candidate except PI3K92 E for which n = 3).

Co-immunoprecipitation (IP) experiments were used to validate the interaction between miPEP8 and protein candidates such as Gcn2 (general control nonderepressible 2), Atg4a (autophagy related gene 4a), PI3K92 E (Phosphatidylinositol 3-kinase 92E) and RagC-D (Ras-related GTP binding C/D) (Fig. 4*A*). S2 cells were co-transfected with plasmids encoding these candidates fused with a HA tag and either the EGFP or EGFP-miPEP8 proteins. Immunoprecipitation using GFP-Trap agarose beads (Chromotek) was performed followed by Western blot analysis. For all the candidates tested a signal was observed in the EGFP-miPEP8 IP but not in the EGFP IP, confirming that all these proteins indeed interact with miPEP8 (Fig. 4*A*). GAPDH-HA (glyceraldehyde-3-phosphate dehydrogenase) was used as negative control to ensure that miPEP8 was not binding its partners in a non-specific manner. Contrary to the other candidates tested, we could not detect any interaction between miPEP8 and GAPDH, strengthening our interactome data (Fig. 4*A*). As a complementary approach, we used split luciferase complementation assays to validate some of the interaction observed by MS (Fig. 4*B*). In this assay, two interacting candidates (here miPEP8 and one of its interacting partners) are both fused to a different fragment of the firefly luciferase (typically referred as N-terminal and C-terminal fragments) (59). If the interacting candidates bind, the fragments of the firefly luciferase are brought into proximity, thus enabling the reconstitution of the enzyme and detection of a luminescent signal can be observed upon addition of its substrate luciferin (59). One of the advantages of protein-fragment complementation assays over co-immunoprecipitation experiments is that the distance required for the fragment of the reporter protein (here the firefly luciferase) needs to be short (inferior to 100 Å) meaning that the candidate interacting proteins are in close proximity and thus this assay reports direct or very close interactions (59). Here, miPEP8 and the candidates were fused to the N-terminal fragment or the C-terminal fragment of the firefly luciferase, respectively (Fig. 4*B*). Each construction encoding the candidates fused to the C-terminal fragment of the firefly luciferase was co-transfected in S2 cells with the construction encoding miPEP8 fused to the N-terminal fragment of the reporter enzyme. In order to check that both fragments of the firefly luciferase did not interact on their own in our experimental conditions, plasmids encoding each fragment not fused to any protein were co-transfected (Fig. 4*B*). Upon lysis of the cells and addition of luciferin, a low luminescence signal was measured (Fig. 4*B*). On the contrary, as a positive control of interaction, the proteins Eyeless (Ey) and Antennapedia (Antp) were fused to N-terminal fragment and the C-terminal fragment of the firefly luciferase, respectively (Fig. 4*B*). These two proteins were previously shown to interact (60). After lysis of the cells and addition of luciferin, a luminescence signal 14.5 times higher than that of the negative control was measured (*p* = 1.7E-03) (Fig. 4*B*) validating our split luciferase approach. Looking at the candidates, luminescence signals were observed for all the proteins tested with a minimal signal 4.6 times higher than the negative control in the case of PI3K92 E (*p* = 3.17E-07) (Fig. 4*B*). Overall, these experiments confirm that miPEP8 interacts with several proteins involved in intracellular signaling pathways and autophagy (Figs. 3, *B* and *C* and 4, *A* and *B*).

### miPEP8 Bears a Short Linear Motif Responsible for its interaction With ref(2)P/p62

**Fig. 5:**
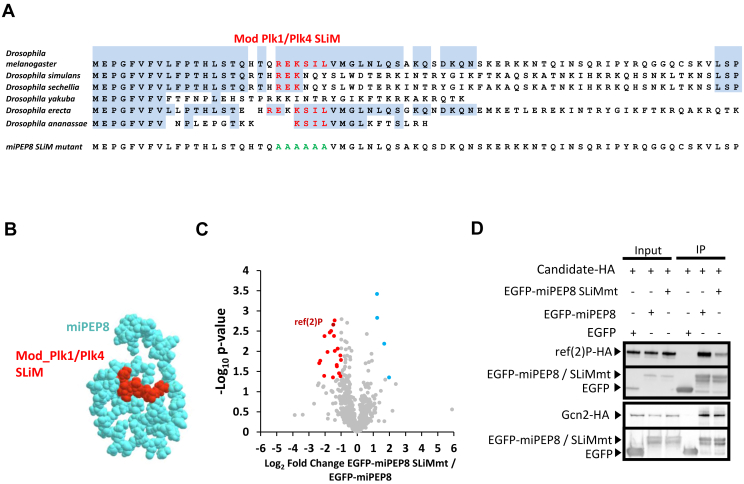
**A MOD_Plk1/MOD_Plk4 SLiM on miPEP8 is involved in its interaction with the protein ref(2)P/p62.***A*, amino acid sequence of miPEP8 and its mutant. The sequences corresponding to the MOD_Plk1/MOD_Plk4 SLiM and the resulting sequence in the mutant (miPEP8 SLiMmt) are shown in *red*. *B*, predicted structure of miPEP8 generated using RoseTTAFold2. *C*, volcano plot representing the log_2_ ratio (EGFP-miPEP8 SLiMmt/EGFP-miPEP8) for each protein quantified in the EGFP-miPEP8 SLiMmt or EGFP-miPEP8 immunoprecipitations (IPs) and the corresponding *p*-value obtained from a Welch's *t* test (n = 4). The *blue*, *red*, and *gray dots* represent the proteins more abundant in the EGFP-miPEP8 IP, more abundant in EGFP-miPEP8 SLiMmt IP, or not differentially associated between the two conditions, respectively. The proteins ref(2)P/p62 and Gcn2 are displayed in *dark blue* on the volcano plot. *D*, co-immunoprecipitation experiments were performed after lysis of S2 cells transfected with plasmids encoding ref(2)P-HA or Gcn2-HA and plasmids encoding either EGFP, EGFP-miPEP8 or EGFP-miPEP8 SLiMmt (n = 3). Western blot analysis for HA and GFP were performed.

To better understand how miPEP8 functions at the molecular level, we looked for the presence of potential protein domains on its sequence using InterProScan (51). Aside from a predicted disordered region spanning from the amino acid 36 to 71, no protein domain could be identified in the sequence of miPEP8. The presence of Short Linear Motifs (SLiMs) was then investigated. SLiMs are short amino acid sequences generally found in disordered regions and involved in protein-protein interactions (61). Using the ELM database SLiM prediction, several potential short motifs were identified on the sequence of miPEP8 (Supplemental Fig. S5*A*). Based on the SLiMs predicted on the miPEP8 sequence, interactions between miPEP8 and canonical proteins from *D. melanogaster* were inferred using the mimicINT workflow (49). The predicted and experimental interactors (based on our interactome data) were compared and a list of 19 proteins emerged as found in both approaches (Supplemental Fig. S5*B*). All these 19 proteins were protein kinases (Supplemental Fig. S5, *B* and *C*) and were predicted to bind the MOD_Plk1/MOD_Plk4 SLiM spanning from the residues 21 to 26 on miPEP8 sequence (Fig. 5*A* and Supplemental Fig. S5*A*). This SLiM motif is partially (mainly the lysine residue) conserved among *Drosophila* species (Fig. 5*A*). Structural prediction of miPEP8 using RoseTTAFold2 (62) showed a central position of this SLiM within this microprotein (Fig. 5*B*). Despite the fact that the MOD_Plk1/MOD_Plk4 SLiM is supposed to be a predicted phosphorylation site on the serine 24 of miPEP8, we could not observe any phosphorylation on this amino acid in our MS data, only the non-phosphorylated serine 24 was observed (Supplemental Fig. S6). We then decided to monitor the effect of the mutation of this SLiM motif (replaced by an alanine stretchFigure 5A) on the interactions between miPEP8 and its partners. An affinity purification followed by MS analysis was performed using the GFP-Trap agarose beads (Chromotek) as described above but S2 cells were transfected with either EGFP, EGFP-miPEP8 or EGFP-miPEP8 SLiMmt (mutated SLiM MOD_Plk1/MOD_Plk4). The mutation of the MOD_Plk1/MOD_Plk4 SLiM changed the interaction of 26 proteins with miPEP8 (Fig. 5*C* and Supplemental Table S4). Among the proteins differentially interacting, one stood out, ref(2)P, the *Drosophila* homolog of the human protein p62/sequestosome-1, involved in autophagy (55) and found upregulated in the proteome of S2 cells upon overexpression of miPEP8 in S2 cells (Fig. 2, *B*–*D*). Upon mutation of the MOD_Plk1/MOD_Plk4 SLiM, the interaction between miPEP8 and ref(2)P/p62 decreased by a 2.8 fold (*p* = 0.0022) (Fig. 5*C* and Supplemental Table S4). This change in interaction was confirmed by western-blot analysis of a co-immunoprecipitation experiment in which S2 cells were co-transfected with ref(2)P-HA and either EGFP, EGFP-miPEP8 or EGFP-miPEP8 SLiMmt (Fig. 5*D*). In parallel, we also co-transfected another interactant of miPEP8, Gcn2 (Figs. 3*B*, 4, *A* and *B* and supplemental Table S3), and either EGFP, EGFP-miPEP8 or EGFP-miPEP8 SLiMmt followed by co-immunoprecipitation and western-blot analysis (Fig. 5*D*). The mutation on the SLiM did not impair the interaction between miPEP8 and Gcn2, confirming that the MOD_Plk1/MOD_Plk4 SLiM is involved in the interaction with only a subset of proteins (Fig. 5*D*).

### Mutation of the MOD_Plk1/Plk4 SLiM on miPEP8 or Knocking Down ref(2)P/p62 Expression Reverse the Decreased of S2 Cell Size Observed Upon Overexpression of miPEP8

**Fig. 6:**
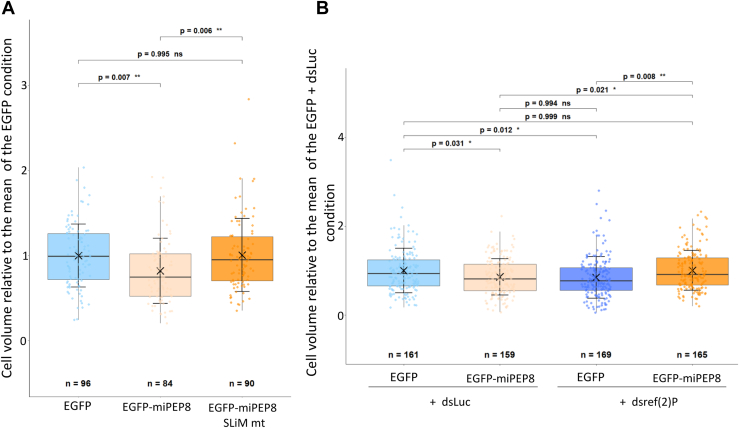
**Mutation of the MOD_Plk1/MOD_Plk4 SLiM on miPEP8 or knocking down ref(2)P expression reverse the reduction of S2 cell size observed upon overexpression of miPEP8.***A*, the size of the S2 cells expressing either EGFP, EGFP-miPEP8 or EGFP-miPEP8 SLiMmt was measured and normalized to the mean of the measured EGFP cell size (n = 96, 84 and 90 for EGFP, EGFP-miPEP8 and EGFP-miPEP8 SLiMmt expressing cells, respectively, from 3 independent experiments). *B*, the size of the S2 cells expressing either EGFP or EGFP-miPEP8 and treated with either Luciferase (dsLuc) or ref(2)P (dsref(2)P) dsRNAs (RNA interference) was measured and normalized to the mean of the measured EGFP dsLuc cell size. The *p*-values for (*A* and *B*) were calculated using a one-way ANOVA (Analysis of Variance) and for groups comparison a Tukey's HSD (Honestly Significant Difference) test was performed (n = 161, 159, 169 and 165 for EGFP dsLuc, EGFP-miPEP8 dsLuc, EGFP dsref(2)P and EGFP-miPEP8 dsref(2)P expressing cells, respectively, from 3 independent experiments).

The effect of the mutation of the MOD_Plk1/MOD_Plk4 SLiM on miPEP8 on cell size was then investigated to see if this motif is involved in the reduction of S2 cell size observed upon overexpression of miPEP8 (Fig. 1, *A* and *B*). S2 cells were transfected with either EGFP, EGFP-miPEP8 or EGFP-miPEP8 SLiMmt (Fig. 6*A*). As expected, a decreased size of S2 cells expressing EGFP-miPEP8 was observed compared to cells expressing EGFP (*p* = 0.007) (Fig. 6*A*). However, cells transfected with EGFP-miPEP8 SLiMmt had a similar size to EGFP transfected cells (*p* = 0.99) and were bigger than cells expressing EGFP-miPEP8 (*p* = 0.006) (Fig. 6*A*). This result points towards a role of the MOD_Plk1/MOD_Plk4 SLiM on miPEP8 on the decreased cell size observed upon overexpression of this microprotein. We then decided to monitor if proteins that are interacting with miPEP8 through this motif were also involved in the phenotype smaller cells. The protein ref(2)P was first investigated as it seems to interact with miPEP8 via its MOD_Plk1/MOD_Plk4 SLiM (Fig. 5, *C* and *D*) and was found upregulated in cells overexpressing miPEP8 (Fig. 2, *B*–*D*). Double-stranded RNA (dsRNA) targeting either the RNA of luciferase protein (dsLuc, negative control) or the RNA of ref(2)P (dsref(2)P) through RNA interference were added to the culture medium of S2 cells for 72 h. Cells were then transfected with either EGFP or EGFP-miPEP8 and the cell size of EGFP positive cells was measured. We controlled that an important decrease of the mRNA level of ref(2)P was observed upon treatment with its dsRNA (Supplemental Fig. S7). Despite the presence of dsLuc, overexpression of EGFP-miPEP8 still induced a decrease in cell size compared to EGFP (*p* = 0.03), showing that application of a control dsRNA does not affect the miPEP8-induced phenotype (Fig. 6*B*). The dsref(2)P also induced a significant reduction of EGFP transfected cell size compared to cells treated with dsLuc (*p* = 0.012) (Fig. 6*B*). Surprisingly, despite the fact that both overexpression of miPEP8 and silencing of ref(2)P result in a reduction of the size of S2 cells, the combination of the expression of the microprotein and down-regulation of ref(2)P reversed the observed phenotype back to control level (EGFP dsLuc, *p* = 0.99) (Fig. 6*B*). Such reversal could not be observed when using dsRNA targeting another protein, RagC-D (Supplemental Fig. S8, *A* and *B*), another protein interacting with miPEP8 (Figs. 3*B*, 4, *A* and *B*, and Supplemental Table S3), despite the fact that RagC-D silencing also seems to decrease cell size (*p* < 0.001). Overall, these results indicate that miPEP8 interacts with ref(2)P and controls S2 cell size through this interaction.

As ref(2)P is involved in autophagy, we decided to monitor the effect of an overexpression of miPEP8 on the autophagic flux. We used the LysoTracker to assess the presence of lysosomes as previously described (25). A significant decrease in the intensity of the signal could be noticed between cells expressing EGFP-miPEP8 versus cells expressing EGFP (Supplemental Fig. S9). We then tested whether this decrease of autophagic flux depends on the MOD_Plk1/MOD_Plk4 SLiM. The LysoTracker signal observed in cells transfected with EGFP-miPEP8 SLiMmt was higher than the one observed in cell overexpressing EGFP-miPEP8 (Supplemental Fig. S9). Thus, the mutation of the MOD_Plk1/MOD_Plk4 SLiM partially reversed the decreased autophagic flux observed following overexpression of EGFP-miPEP8. This result indicates that the modulation of the autophagic flux induced by the overexpression of miPEP8 seems to be linked to the concomitant increase of the protein level of ref(2)P (Fig. 2, *B*–*D*) and in a mechanism dependent of its MOD_Plk1/MOD_Plk4 SLiM.

As several proteins from the mTOR signaling pathway, which is known to regulate autophagy, were found in the interactome of miPEP8, we monitored the effect of Torin 1 on the cell size phenotype mediated by the overexpression of EGFP-miPEP8 (Supplemental Fig. S10). Interestingly, the addition of Torin 1 reversed the decrease of cell size induced by EGFP-miPEP8 overexpression (Supplemental Fig. S10), suggesting a role of the mTOR signaling pathway in the function of miPEP8.

### Mutation or Overexpression of miPEP8 in Flies Decreases Cell Size

**Fig. 7:**
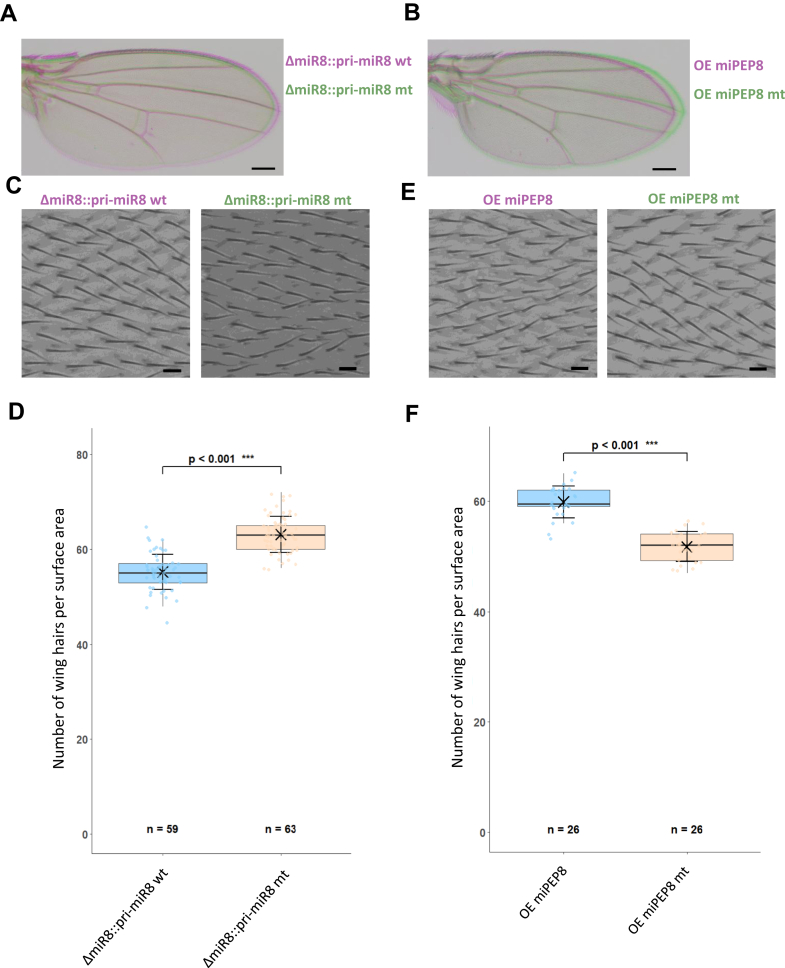
**Mutation or overexpression of miPEP8 in flies decreases wing cell size.***A*, representative pictures of wing from flies expressing either a wt primiR8 rescued construct (ΔmiR8::pri-miR8 wt, *pink*) or a miPEP8 mutated primiR8 rescue construct (ΔmiR8::pri-miR8 mt, *green*) (bar = 200 μm). The pri-miR-8 miPEP-8 mutated (untranslatable miPEP8) shows a reduced wing compared to the wild type pri-miR-8 (n = 3). *B*, representative pictures of wing of flies overexpressing either miPEP8 (OE miPEP8, *pink*) and an untranslatable miPEP8 mutated line (OE miPEP8 mt, *green*) fly (bar = 200 μm). Overexpression of miPEP8 induced a reduced wing compared to the control line expressing the same construct unable to translate miPEP8 (n = 3). Expression of miPEP8 in wing was directed by the MS1096-GAL4 driver (27). *C*, magnification of the wing L3-L4 intervein region showing wing hairs (one hair/cell) from flies expressing either a wt primiR8 rescued construct (ΔmiR8::pri-miR8 wt) or a miPEP8 mutated primiR8 rescue construct (ΔmiR8::pri-miR8 mt) (bar = 10 μm). *D*, quantification of the number of hair/cells in 0.01 mm^2^ wing areas from flies from flies expressing either a wt primiR8 rescued construct (ΔmiR8::pri-miR8 wt) or a miPEP8 mutated primiR8 rescue construct (ΔmiR8::pri-miR8 mt). The statistical significance was calculated using a student's *t* test. *E*, magnification of the wing L3-L4 intervein region showing wing hairs (one hair/cell) from flies overexpressing either miPEP8 (OE miPEP8) and an untranslatable miPEP8 mutated line (OE miPEP8 mt) (bar = 10 μm). Expression of miPEP8 in wing was directed by the MS1096-GAL4 driver (27). *F*, quantification of the number of hair/cells in 0.01 mm^2^ wing areas from flies from flies overexpressing either miPEP8 (OE miPEP8) and an untranslatable miPEP8 mutated line (OE miPEP8 mt). The statistical significance was calculated using a student's *t* test (n for each condition is indicated on the graph).

In order to see if the cell size phenotype we observed in S2 cells upon EGFP-miPEP8 overexpression (Fig. 1, *A* and *B*) could be recapitulated *in vivo*, we took advantage of the fact that *Drosophila* wing epidermal cells each produces a single cuticular hair. This model was frequently used to monitor the number and/or the size of cells by counting the number of hairs (representative of the number of cells) within a precise surface (33, 52, 53). Less hairs (cells) per surface indicate that wing size reduction is due to reduction of the number of cells, whereas more hairs (cells) indicate that cells have a reduced size. Therefore, the numbers of hairs per surface area between the L3 and L4 veins were counted in flies mutated for or overexpressing miPEP8. We first confirmed that the mutation (Fig. 7*A*) or overexpression (Fig. 7*B*) of miPEP8 induced a reduction in wing size as previously described (28). Counting the cuticular hairs per surface area showed that flies mutated for miPEP8 have smaller cell sizes than wild type flies (Fig. 7, *C*–*E*) (*p* < 0.001). Such difference in cell size was also observed in flies overexpressing miPEP8 in wings using the MS1096 driver compared to flies overexpressing the same construction but with a mutated initiation codon (Fig. 7, *F*–*H*) (*p* < 0.001). These *in vivo* data confirm that miPEP8 is involved in the regulation of cell size in *D. melanogaster*.

## Conclusion

As it has recently been shown, microproteins seem to have a wide range of functions in the development and physiology (fitness) of *D. melanogaster* (25, 26). A previous study from our group identified a microprotein encoded by the *miR-8* gene that is involved in fly survival and wing size regulation (28). Here, we investigated how miPEP8 functions at the molecular level. Overexpression of this microprotein in S2 cells showed a decrease of cell size and alteration of the cell cycle (Fig. 1 and Supplemental Fig. S2). This is consistent with the phenotype of flies mutated or overexpressing miPEP8, which have smaller cells on their wings (Fig. 7) and a reduced allelic frequency ratio, indicating reduced survival capacity (28). Here, we show that the reduction of cell size seems to be mediated through the interaction between miPEP8 and ref(2)P/p62 via a SLiM located on miPEP8 as knocking down ref(2)P/p62 or mutating the SLiM on miPEP8 brought back the cells to their original sizes (Fig. 6). In addition to the decrease of cell size, overexpression of miPEP8 in S2 cells also accumulated cells in the G1 phase of the cell cycle and decreases the autophagic flux (Supplemental Figs. S2 and S9). The latter seems to be at least partially mediated through the MOD_Plk1/MOD_Plk4 SLiM and thus depending on the interaction between miPEP8 and ref(2)P/p62 (Supplemental Fig. S9). It is not clear however what are the molecular mechanisms involved in the interaction between miPEP8 and ref(2)P/p62 and how the overexpression of miPEP8 modulated ref(2)P/p62 protein level and/or activity. Our experiments point towards a post transcriptional regulation of ref(2)P/p62 (Supplemental Fig. S4) upon overexpression of miPEP8. Increased protein level of ref(2)P/p62 is associated to a decrease of the autophagic flux, consistent with our LysoTracker data (Supplemental Fig. S9). One might hypothesize that the binding of miPEP8 could impair the formation of ref(2)P/p62 oligomers or prevent the interaction between ref(2)P/p62 and Atg8a/LC3, affecting in either cases selective autophagy and thus preventing ref(2)P/p62 degradation by the lysosome (63, 64). However, ref(2)P/p62 is involved in many processes beyond autophagy (64) and further experiments would be needed to clarify these points. Among the SLiMs identified on miPEP8, one of them in the N-terminal part of the microprotein is a LIG_LIR_Gen_1 which is involved in the binding of proteins to the forming autophagosome and well conserved among *Drosophila* species (Fig. 5*A* and Supplemental Fig. S5). In addition, the protein level of Atg18b was found upregulated upon miPEP8 overexpression and Atg4a was identified as a protein partner of miPEP8 (Figs. 3*B* and 4, *A* and *B*). Both of these proteins are involved in autophagosome formation (65, 66). It would be interesting to see if these two proteins and the LIG_LIR_Gen_1 identified on miPEP8 are also involved in the decreased autophagic flux observed upon overexpression of miPEP8.

Besides its role in regulating cell size and autophagy, miPEP8 might have other function in *Drosophila*. Indeed, despite the use of stringent criteria to identify proteins associated to miPEP8 in our interactome data, more than 200 proteins were found interacting with this microprotein (Fig. 3*B*). Although we cannot exclude that some of these interactions might be due to the overexpression of miPEP8, it is quite surprising to observe many protein kinases associated to this small protein (Fig. 3*B* and Supplemental Table S3). Our data might point to a function of miPEP8 in the regulation of key signaling pathways such as the PI3K/Akt/mTORC1 or MAPK pathways (Fig. 3*B*). Importantly, a treatment with Torin 1 reversed the small cell size phenotype observed upon EGFP-miPEP8 overexpression (Supplemental Fig. S10) suggesting that the mTOR pathway might be regulating miPEP8 in some ways. Recent studies have suggested that many microprotein-encoding genes play a role in organismal fitness, as revealed by weak developmental defects and detectable phenotypes that only appear when organisms are challenged (25, 26). One possible explanation is that microproteins are modulators of cellular pathways. Our results support this hypothesis, as neither the gain-of-function nor the loss-of-function of miPEP8 induces strong developmental defects. Moreover, the interactors of miPEP8 identified are components of pathways involved in cell survival, fitness, and stress response (Fig. 3, *B* and *C*). All in all, the present study provides a first molecular insight into the function of miPEP8 in *Drosophila* and precises its role in the regulation of cell size through an interaction with ref(2)P, as well as paving the way to future study of the involvement of this microprotein in other cellular pathways.

## Data Availability

All the mass spectrometry data have been deposited with the MassIVE repository with the dataset identifier: MSV000098269.

## Supplemental data

This article contains supplemental data.

## Conflict of interest

The authors declare that they do not have any conflicts of interest with the content of this article.
